# The Crystal Structure of Micro- and Nanopowders of ZnS Studied by EPR of Mn^2+^ and XRD

**DOI:** 10.1186/s11671-016-1739-4

**Published:** 2016-11-24

**Authors:** Valentyna Nosenko, Igor Vorona, Valentyn Grachev, Stanislav Ishchenko, Nikolai Baran, Yurii Becherikov, Anton Zhuk, Yuliya Polishchuk, Vasyl Kladko, Alexander Selishchev

**Affiliations:** 1 V. Lashkaryov Institute of Semiconductor Physics of National Academy of Sciences of Ukraine, 45, Pr. Nauky, Kyiv, 03028 Ukraine; 2 Physics Department, Montana State University, Bozeman, MT 59717 USA; 3 L.V. Pisarzhevskii Institute of Physical Chemistry, National Academy of Sciences of Ukraine, 31, Pr. Nauky, Kyiv, 03028 Ukraine

**Keywords:** ZnS, EPR, XRD, Paramagnetic defect, Manganese ions, Lattice structure

## Abstract

The crystal structure of micro- and nanopowders of ZnS doped with different impurities was analyzed by the electron paramagnetic resonance (EPR) of Mn^2+^ and XRD methods. The powders of ZnS:Cu, ZnS:Mn, ZnS:Co, and ZnS:Eu with the particle sizes of 5–7 μm, 50–200 nm, 7–10 μm, and 5–7 nm, respectively, were studied. Manganese was incorporated in the crystal lattice of all the samples as uncontrolled impurity or by doping. The Mn^2+^ ions were used as EPR structural probes. It is found that the ZnS:Cu has the cubic structure, the ZnS:Mn has the hexagonal structure with a rhombic distortion, the ZnS:Co is the mixture of the cubic and hexagonal phases in the ratio of 1:10, and the ZnS:Eu has the cubic structure and a distorted cubic structure with stacking defects in the ratio 3:1. The EPR technique is shown to be a powerful tool in the determination of the crystal structure for mixed-polytype ZnS powders and powders with small nanoparticles. It allows observation of the stacking defects, which is revealed in the XRD spectra.

## Background

The interest to zinc sulfide has recently increased due to the progress in the technologies that allow the obtaining of low-sized (LS) ZnS crystals on the scale from micrometer to a few nanometer. LS ZnS demonstrates new properties that are interesting from the point of view of both fundamental and applied physics. In particular, these are the blue shift of the fundamental absorption band for the LS ZnS [1, 2], decrease of the zinc blende-to-wurtzite (cubic-to-hexagonal) phase transition temperature [3, 4], and increase of efficiency of the photoluminescence [5] and low-voltage cathodoluminescence [6]. LS ZnS can be applied in various fields, such as fabrication of new materials for scintillation detectors [7–9], thermoluminescence dosimetry [10], solar cells [10], and light-emitting sources [11].

Changes of the LS ZnS crystals properties are caused both by their small sizes and specific features of their local structure, in particular, by local distortions of the crystalline lattice in the vicinity of impurity centers. The latter alters the properties of the centers or even transforms them into new ones. For example, paramagnetic Mn^2+^ centers located near planar stacking defects and affected by local axial crystal field distortions were detected in the LS cubic ZnS:Mn [12]. Surface Mn^2+^ centers were also observed [13–15].

To obtain information about the structure of the crystal lattice the X-ray diffraction (XRD) analysis is usually used. However, at the reducing of the crystal size to a few nanometers and the presence of mixtures of crystalline phases, the XRD results are not always unambiguous. XRD also does not detect local distortions of the lattice, in particular, near to the impurities. In these cases, the electron paramagnetic resonance (EPR) is more effective, especially, when paramagnetic centers with high spin serve as structure probes. An example of such high spin centers is Mn^2+^ ion, which has the electron spin *S* = 5/2 and the nuclear spin *I* = 5/2 and is extremely sensitive to the structure of the nearest surroundings. Note also the favorable fact that the manganese in sufficient amount for observation in the EPR spectra often presents even in nominally “pure” materials as uncontrolled impurities. Moreover, materials specially doped with manganese are often being of interest.

However, it should be noted that to obtain the above-mentioned structural information by the EPR method, the detailed description of the EPR spectra using the spin-Hamiltonian (SH) and determination of all its parameters are necessary. In the case of low-dimension powder materials, it is a challenge. Difficulties are caused by the spatial averaging of the complex spectra with allowed and forbidden transitions, as well as by the EPR line broadening due to significant distortion in the crystal lattice of low-sized materials that varies from particle to particle [14]. These factors cause the lineshape change and even smoothing of the resonance lines. Therefore, the determination of the SH parameters, that give the information about the local crystal structure, becomes more complicated. In this paper, these difficulties have been overcome by the usage of the high-precision computer program for EPR spectra description.

The results of the XRD and EPR study of the crystal structure of the LS ZnS powders doped with Cu, Co, Mn, and Eu are present below.

## Methods

The zinc sulfide powders prepared for various optoelectronic applications were used. The samples of ZnS:Cu, ZnS:Co, and ZnS:Mn were obtained by the self-propagating high-temperature synthesis (SHS) [16]. The samples were doped by copper, cobalt, or manganese during the synthesis by adding of the corresponding salts in the charge. The contents of the impurities in the charge was 1 wt%. As a result of the synthesis, powders of ZnS:Cu, ZnS:Mn, and ZnS:Co were obtained with the particle sizes of 5–7 μm, 50–200 nm, and 7–10 μm, respectively. Manganese was not introduced in the ZnS:Cu and ZnS:Co powders especially because it was present in these samples as an uncontrolled impurity. The measurements were carried out after 4–5 months of storage needed to complete structure relaxation of the powders obtained under non-equilibrium SHS conditions. The ZnS:Eu nanopowders were synthesized from the colloidal solution. The methods of joint and successive decomposition of zinc(II) and europium(III) diethyldithiocarbamate complexes in the mixture of high boiling point solvents oleylamine and 1-octadecene at 240 °C temperature were used. The molar ratio of Eu:Zn in the solution was 1%. The detailed description of the synthesis is in [17]. According to TEM, the particle sizes of the zinc sulfide powders synthesized by the joint and successive decomposition methods were 5–7 nm. Manganese was present in the samples as an uncontrolled impurity. Note that the X-ray fluorescence analysis showed dopants in the range of 0.1–0.2% for all the samples.

The XRD studies were carried out on the powder diffractometer Thermo Techno ARL X’TRA in Bragg geometry. Data were collected in 2*θ* range 20–80°, with the step 0.02°. The measurements were carried out at room temperature (wavelength Kα1 = 1.540562 Å). For high-quality spectrum analysis, the Search Match software package was used. For the quantitative analysis, the Jana 2006 software [18] was used.

The EPR measurements were carried out using X-band spectrometer Varian E12 at room temperature. One hundred-kilohertz modulation of the magnetic field and the microwave power of below 2 mW (that is much less than the saturation power) were applied. Simulation of EPR spectra was carried out using computer program “POWDER” from the “Visual EPR” software package [19], based on the algorithm described in [20].

## Results and Discussion

The manganese ions, being incorporated into the ZnS lattice, can substitute for Zn^2+^ ions and form Mn^2+^ centers. To describe the EPR spectrum of the Mn^2+^ center, the SH containing the electronic and nuclear Zeeman interactions, the hyperfine interaction, and the interaction of the electron spin with the crystal field (zero field splitting term) is usually used:

$$ H=g\beta \mathbf{B}\mathbf{S}-{g}_{\mathrm{N}}{\beta}_{\mathrm{N}}\mathbf{BI}+A\mathbf{S}\mathbf{I}+{\displaystyle \sum_{n.m}{f}_n{b}_n^m{O}_n^m}. $$

Here, the standard designations (see, for example, [21, 22]) are used. The values of *g*, *g*_N_, *β*, *β*_N_, and *A* are assumed to be isotropic. The set of *b*_n_^m^ parameters is determined by the surroundings of the Mn^2+^ and depends on the type of ZnS lattice and its distortion. The Mn^2+^ centers were extensively studied in bulk zinc sulfide single crystals. In the cubic crystal lattice, the EPR spectrum of the Mn^2+^ centers is described by following SH parameters *g* = 2.00225 ± 0.00006, *a* = (7.987 ± 0.008) × 10^−4^ cm^−1^, and *A* = −(63.88 ± 0.02) × 10^−4^ cm^−1^ [23]. In the hexagonal lattice, the SH parameters are *g* = 2.0016, *A* = −65 × 10^−4^ cm^−1^, *b*_2_^0^ = −105 × 10^−4^ cm^−1^, 3*b*_4_^0^ − 0.1*b*_4_^3^ = 7.6 × 10^−4^ cm^−1^ [24], or *g* = 2.0018, *A* = −64.9 × 10^−4^ cm^−1^, *b*_2_^0^ = −130.9 × 10^−4^ cm^−1^, *b*_4_^0^ = 3.9 × 10^−4^ cm^−1^, and *b*_4_^3^ = 70 × 10^−4^ cm^−1^ [25]. In low-sized ZnS, a distortion of the crystal lattice and/or changing of the center localization are possible. These effects should alter the SH parameters. To optimize the computer simulation of experimental spectra, we started with the rough estimation of the dominating parameters in the SH. For this purpose, the experimental spectra were compared with a set of model Mn^2+^ EPR spectra calculated for powders with the program [19] for different values of parameter *b*_n_^m^. Note that in the powder, EPR spectrum of the Mn^2+^ dominates (has the highest intensity) of a set of six hyperfine lines corresponding to the central electronic transition +1/2↔−1/2. Other fine lines caused by the electron transitions ±3/2↔ ± 1/2 and ±5/2↔ ± 3/2 (non-central transitions) for *b*_n_^m^ ≠ 0 are weaker due to its spatial averaging. Therefore, as a typical example, we mention the characteristic influence of the crystal fields with different symmetry on the intense lines sextet of the central transition. Thus, when *b*_4_^0^ prevails among the SH parameters (cubic structure), the sextet lines have equal intensity. In the case of the predominance of *b*_2_^0^, which is characteristic of the hexagonal lattice, each sextet line has the doublet splitting increasing with the magnetic field. These features allow to estimate the dominate crystal lattice in the powders. Lowering the crystal field symmetry reduces the relative intensities of the non-central transitions. The final determination of the SH parameters of the Mn^2+^ centers was carried out both by varying the values of the dominant SH parameters and by introducing additional parameters, which reflect the local lattice distortions. The attention was also paid to the detailed description of forbidden transitions. Possible distribution of the SH parameters were also taken into account by averaging of the corresponding spectra. Note that the computer programs that use the perturbation theory limited by the second order do not give the correct result.

**Fig. 1:**
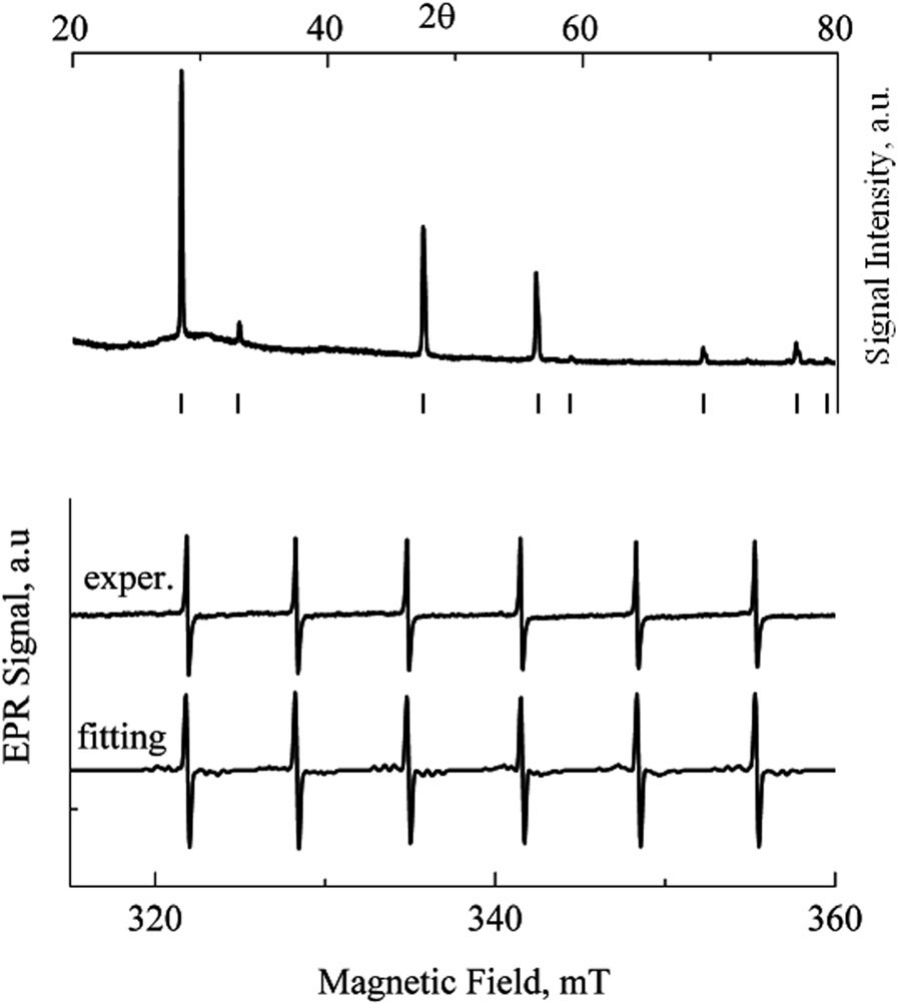
XRD and EPR spectra of Mn^2+^ in ZnS:Cu powder with particle sizes of 5–7 μm

Figure 1 shows the results of the ZnS:Cu powder study. The experimental XRD spectrum is shown in the upper part of the figure, and also, the positions of the cubic zinc sulfide reflexes are shown by dashes. Detailed analysis of the spectrum using Jana 2006 program confirmed that the XRD spectrum is caused only by the cubic phase of the zinc sulfide being free from an admixture of any other phase. The lower part of Fig. 1 demonstrates the experimental and model EPR spectra of the Mn^2+^ in the ZnS:Cu sample containing manganese as an uncontrolled impurity. Preliminary analysis of the experimental EPR spectrum showed dominance of the cubic structure that agrees with the XRD results. The following SH parameters were determined from the experimental spectrum by the program [19]: *g* = 2.0022 ± 0.0002, *A* = (−62.7 ± 0.2) × 10^−4^ cm^−1^, and *b*_4_^0^ ≥ 3.5 × 10^−4^ cm^−1^ (*b*_4_^0^ parameter was estimated from the linewidth Δ*B* = 0.1 mT). The model spectrum calculated using the above parameters has higher intensity of the transitions ±3/2↔ ± 1/2 and ±5/2↔ ± 3/2 compared with the experimental spectrum. Most likely, this is due to the broadening of the lines of these transitions caused by scatter of *b*_4_^0^ constant value in the powder. Good agreement between the experimental and fitting spectrum shows that the powder ZnS:Cu has the cubic structure.

**Fig. 2:**
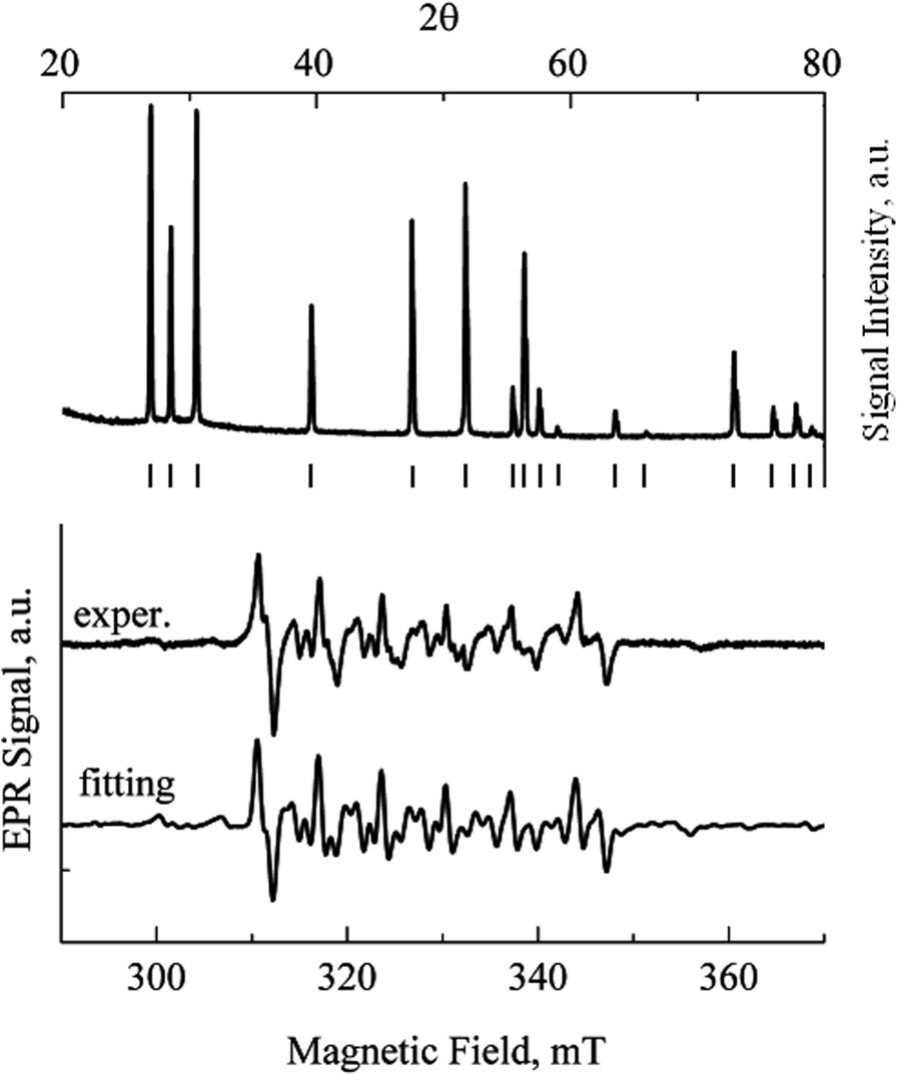
XRD and EPR spectra of Mn^2+^ in ZnS:Mn powder with particle sizes of 50–200 nm

Figure 2 presents the results of the study of the ZnS:Mn powder by the XRD and EPR methods. The XRD spectrum (see the upper part of the Fig. 2) showed that the structure of the powder particles is the hexagonal (2H) modification of the zinc sulfide. The detailed analysis of the XRD spectrum has shown the powder free from an admixture of any other phase. The lower part of Fig. 2 demonstrates the experimental EPR spectrum for the powder. In general outline, it corresponds to the dominance of the hexagonal structure. The best fitting was obtained using the SH parameters *g* = 2.0025 ± 0.0002, *A* = (−62.9 ± 0.2) × 10^−4^ cm^−1^, *b*_2_^0^ = (−105 ± 3) × 10^−4^ cm^−1^, *b*_4_^0^ = 3.5 × 10^−4^ cm^−1^, *b*_4_^3^ = 70 × 10^−4^ cm^−1^, and Δ*B* = 0.7 mT, also taking into account the *b*_2_^2^ parameter (rhombic distortion) and variation of its value ±10^−3^ cm^−1^. The corresponding spectrum averaging was done taking into account this variation.

**Fig. 3:**
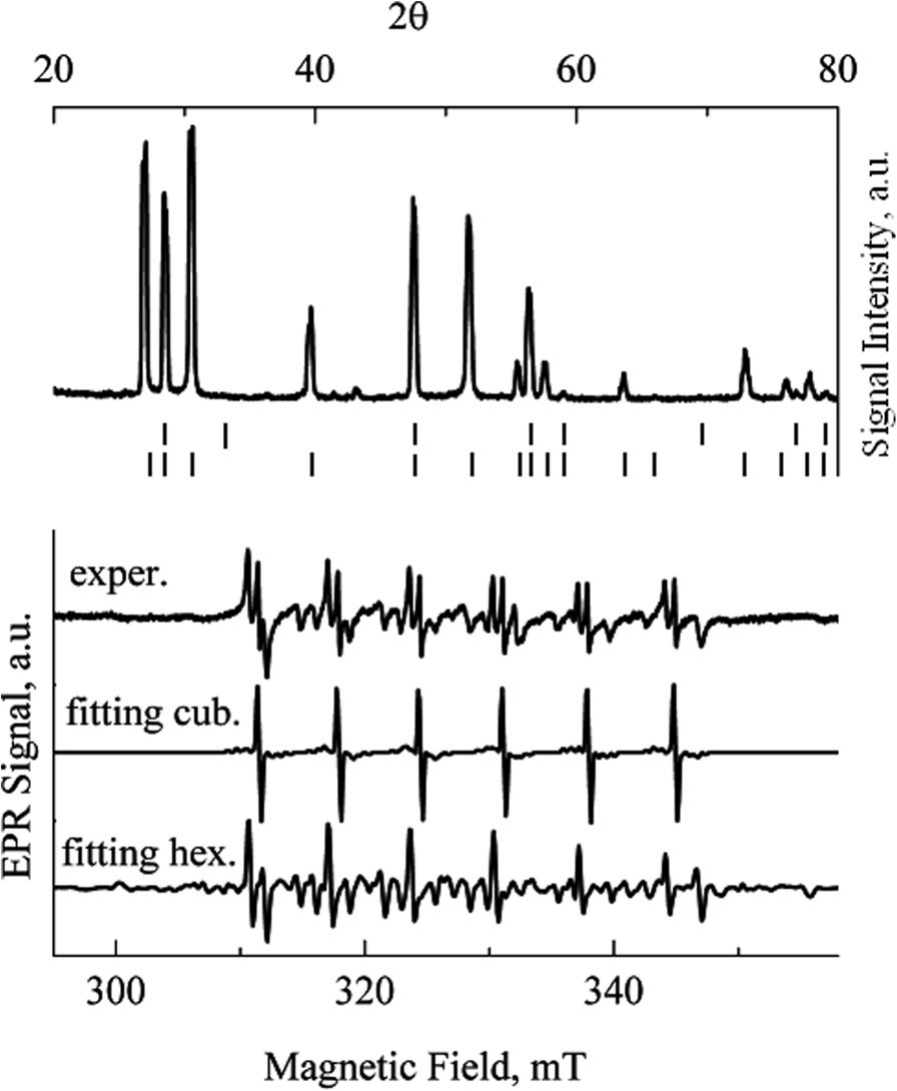
XRD and EPR spectra of Mn^2+^ in ZnS:Co powder with particle sizes of 7–10 μm

Figure 3 demonstrated the XRD (upper part of the Fig. 3) and the EPR (the lower part of the Fig. 3) spectra of the ZnS:Co powder. Qualitative XRD analysis showed that the powder particles have the hexagonal (2H) structure. The detailed analysis of the phase composition using Jana 2006 programs [18] revealed in the sample an admixture of the ZnS cubic phase. It should be noted that the revealing of the cubic phase in this case was fraught with many difficulties and quantitatively is not unambiguous. The experimental EPR spectrum of uncontrolled Mn^2+^ impurity in the ZnS:Co was the mixture of two spectra: Mn^2+^ in the cubic and hexagonal ZnS structures. Therefore, the experimental spectrum was fitted by these two components. The determined SH parameters of Mn^2+^ in the cubic surrounding within the experimental error were equal to the corresponding parameters of the Mn^2+^ ion in the ZnS:Cu. The SH parameters of the Mn^2+^ in the hexagonal structure determined from the best fitting of the experimental spectra are *g* = 2.0025 ± 0.0002, *A* = (−62.9 ± 0.2) × 10^−4^ cm^−1^, *b*_2_^0^ = (−105 ± 3) × 10^−4^ cm^−1^, and Δ*B* = 0.1 mT. To improve fitting, we used the values *b*_4_^0^ = 3.5 × 10^−4^ cm^−1^ and *b*_4_^3^ = 70 × 10^−4^ cm^−1^ estimated in [25]. From the ratio of the integrated intensities of the spectra, it was found that the ZnS:Co sample has the mixture of the cubic and hexagonal phases with the ratio of about 1:10.

**Fig. 4:**
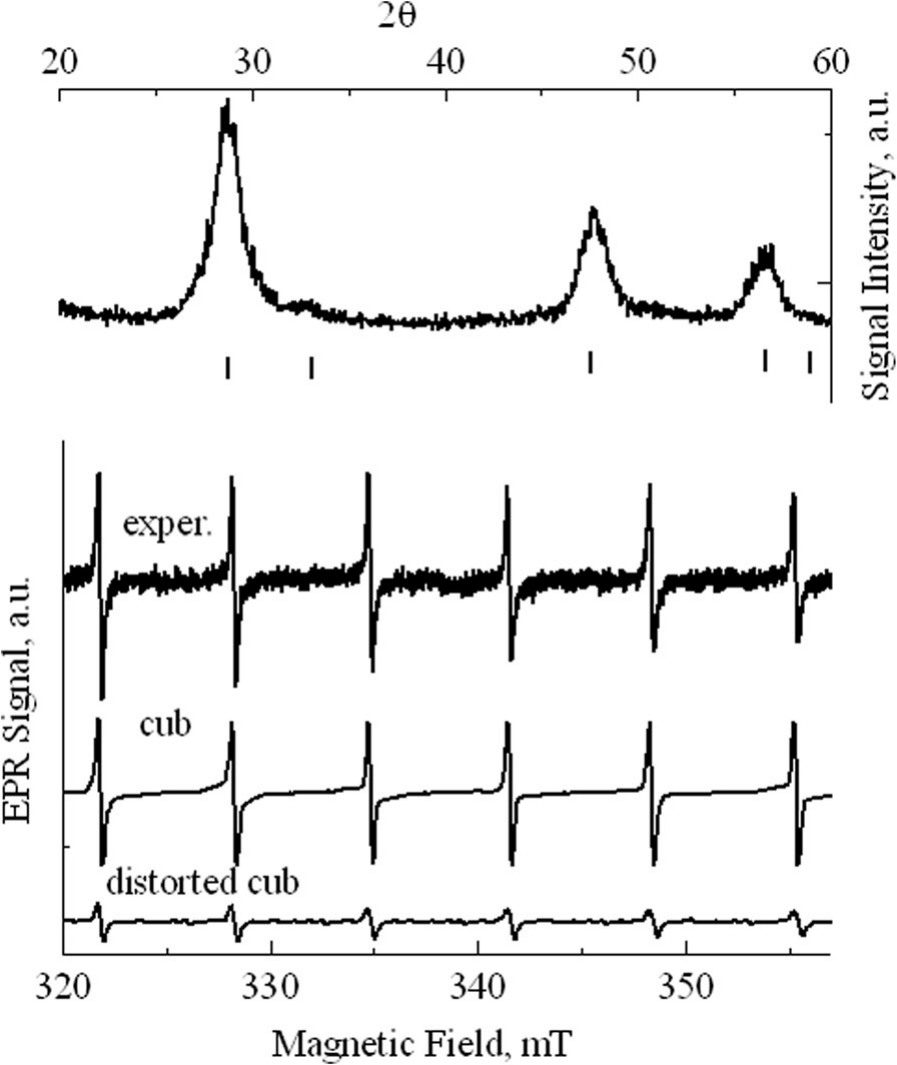
XRD and EPR spectra of Mn^2+^ in ZnS:Eu powder with particle sizes of 6–6.5 nm

The XRD and EPR spectra of the ZnS:Eu nanopowder are shown in Fig. 4. Reflexes in the XRD spectrum (upper part of Fig. 4) are significantly broader compared with the reflexes of previous powders that contain larger particles. However, the XRD spectrum can still be identified with the cubic zinc sulfide. It should be noted that at further reduction of the particle size occurs even more significant broadening of the reflexes (see, for example, [4]), whereby the unambiguous interpretation of the XRD spectra and accordingly the determination of the crystal structure becomes difficult. The EPR spectrum (lower part of Fig. 4) of the ZnS:Eu powder is visually similar to the spectrum of Mn^2+^ ions in the cubic lattice (see Fig. 1). However, its detailed analysis reveals some features. The intensities of lines decrease with increasing of the magnetic field. In addition, there is the appearance of high-field wing in each EPR line, which contribution growths with increasing of the magnetic field. The detailed analysis of the lineshapes of all spectral lines showed that the spectrum can be explained by the presence of two signals. The first one is the signal of Mn^2+^ ions in the cubic lattice with above parameters. The second signal is described by the parameters *g* = 2.0022 ± 0.0002, *A* = (−62.7 ± 0.2) × 10^−4^ cm^−1^, and *b*_2_^0^ = (−36 ± 1) × 10^−4^ cm^−1^. Similar signal has been observed previously in the mixed-polytypes single crystals of the zinc sulfide consisting of the cubic and hexagonal phases [26, 27]. Recently, the signal with similar parameters was observed in the nanocrystalline cubic ZnS, and the model of the center (distorted cubic site) was proposed [12, 28]. According to [12, 28], the Mn^2+^ ion is located in the site of the cubic lattice next to planar stacking defect (the first sphere of surrounding). In studied ZnS:Eu powder, the amount ratio of the “normal cubic” manganese and the “distorted cubic” manganese is approximately 3:1.

## Conclusions

The crystal structure of the low-sized ZnS powders was comparatively examined by the XRD and the Mn^2+^ EPR. For the samples studied, the following results was found. Both XRD and EPR techniques showed that the ZnS:Cu powder with the particle size of 5–7 μm has the cubic structure without an admixture of other phases. The XRD of the ZnS:Mn (50–200 nm) revealed the hexagonal structure of the powder and the Mn^2+^ EPR found the hexagonal structure with a rhombic distortion. The ZnS:Co (7–10 μm) was found to have the hexagonal structure with an admixture of the cubic phase by both the XRD and EPR methods. The XRD detection of the cubic phase in the powders is ambiguous while the EPR allows determining the ratio of the hexagonal and cubic phases being 10: 1. The XRD of the ZnS:Eu (6–6.2 nm) found the cubic structure (unreliable due to the broadening of reflexes) while the EPR detected the regular cubic structure and the cubic structure with planar stacking faults in the ratio of 3:1. Thus, the use of the Mn^2 +^ions EPR provides the more complete and unambiguous information about the structure of micro- and nanozinc sulfide. The Mn^2+^ EPR can serve as the method of the rapid analysis of mixed-polytype zinc sulfide powders and powders and XRD reflections which broadened (particle size less than 5 nm). In addition, the EPR allows to establish the presence of the local distortion of the crystal lattice, which is not available by the XRD.

## Abbreviations

EPR: Electron paramagnetic resonance
LS: Low-sized
SH: Spin-Hamiltonian
XRD: X-ray diffraction
